# RETRACTION: The Impact of Physiotherapy Interventions on Enhancing Wound Healing Post Knee Osteoarthritis Surgery: A Meta‐Analysis

**DOI:** 10.1111/iwj.70584

**Published:** 2025-04-18

## Abstract

**RETRACTION:** YangJ. , ChuR. , ChenZ. , QiuJ. , PangZ. , and YangH. , “The Impact of Physiotherapy Interventions on Enhancing Wound Healing Post Knee Osteoarthritis Surgery: A Meta‐Analysis,” International Wound Journal 21, no. 2 (2024): e14777, 10.1111/iwj.14777.38361227 PMC10869649

The above article, published online on 15 February 2024, in Wiley Online Library (http://onlinelibrary.wiley.com/), has been retracted by agreement between the journal Editor in Chief, Professor Keith Harding; and John Wiley & Sons Ltd. Following an investigation by the publisher, all parties have concluded that this article was accepted solely on the basis of a compromised peer review process. The editors have therefore decided to retract the article. The authors did not respond to our notice regarding the retraction.

**RETRACTION:** J. Yang , R. Chu , Z. Chen , J. Qiu , Z. Pang , and H. Yang , “The Impact of Physiotherapy Interventions on Enhancing Wound Healing Post Knee Osteoarthritis Surgery: A Meta‐Analysis,” International Wound Journal 21, no. 2 (2024): e14777, 10.1111/iwj.14777.38361227 PMC10869649

The above article, published online on 15 February 2024, in Wiley Online Library (http://onlinelibrary.wiley.com/), has been retracted by agreement between the journal Editor in Chief, Professor Keith Harding; and John Wiley & Sons Ltd. Following an investigation by the publisher, all parties have concluded that this article was accepted solely on the basis of a compromised peer review process. The editors have therefore decided to retract the article. The authors did not respond to our notice regarding the retraction.

